# PAC down-regulates estrogen receptor alpha and suppresses epithelial-to-mesenchymal transition in breast cancer cells

**DOI:** 10.1186/s12885-016-2583-8

**Published:** 2016-07-27

**Authors:** Huda A. Al-Howail, Hana A. Hakami, Basem Al-Otaibi, Amer Al-Mazrou, Maha H. Daghestani, Ibrahim Al-Jammaz, Huda H. Al-Khalaf, Abdelilah Aboussekhra

**Affiliations:** 1 Department of Molecular Oncology, King Faisal Specialist Hospital and Research Center, MBC # 03, PO BOX 3354, Riyadh, 11211 Kingdom of Saudi Arabia; 2 Department of Cyclotron and Radiopharmaceuticals, King Faisal Specialist Hospital and Research Center, Riyadh, 11211 Kingdom of Saudi Arabia; 3 Stem Cell Therapy Program, King Faisal Specialist Hospital and Research Center, Riyadh, 11211 Kingdom of Saudi Arabia; 4 Department of Zoology, College of Science, King Saud University, Riyadh, 11451 Kingdom of Saudi Arabia; 5 The National Center for Genomics Research, King Abdulaziz City for Science and Technology, Riyadh, 11211 Kingdom of Saudi Arabia; 6 Present Address: McGill University Health Center, Montreal, QC Canada

**Keywords:** PAC, ERα, Breast cancer, EMT

## Abstract

**Background:**

Triple-negative breast cancer (TNBC) is an aggressive histological subtype with limited treatment options and very poor prognosis following progression after standard chemotherapeutic regimens. Therefore, novel molecules and therapeutic options are urgently needed for this category of patients. Recently, we have identified PAC as a curcumin analogue with potent anti-cancer features.

**Methods:**

HPLC was used to evaluate the stability of PAC and curcumin in PBS and also in circulating blood. Cytotoxicity/apoptosis was assessed in different breast cancer cell lines using propidium iodide/annexinV associated with flow cytometry. Furthermore, immunoblotting analysis determined the effects of PAC on different oncogenic proteins and pathways. Additionally, the real time xCELLigence RTCA technology was applied to investigate the effect of PAC on the cellular proliferation, migration and invasion capacities.

**Results:**

PAC is more stable than curcumin in PBS and in circulating blood. Furthermore, we have shown differential sensitivity of estrogen receptor-alfa positive (ERα^+^) and estrogen receptor alfa negative (ERα^−^) breast cancer cells to PAC, which down-regulated ERα in both cell types. This led to complete disappearance of ERα in ERα^−^ cells, which express very low level of this receptor. Interestingly, specific down-regulation of ERα in receptor positive cells increased the apoptotic response of these cells to PAC, confirming that ERα inhibits PAC-dependent induction of apoptosis, which could be mediated through ERα down-regulation. Additionally, PAC inhibited the proliferation and suppressed the epithelial-to-mesenchymal transition process in breast cancer cells, with higher efficiency on the TNBC subtype. This effect was also observed in vivo on tumor xenografts. Additionally, PAC suppressed the expression/secretion of 2 important cytokines IL-6 and MCP-1, and consequently inhibited the paracrine procarcinogenic effects of breast cancer cells on breast stromal fibroblasts.

**Conclusion:**

These results indicate that PAC could be considered as important candidate for future therapeutic options against the devastating TNBC subtype.

## Background

Despite advances in diagnosis and treatment, breast cancer remains a major public health problem and a major cause of death for women worldwide [1, 2]. Recent gene expression profiling studies have shown that this heterogeneous disease is composed of five major biological subtypes: luminal A, luminal B, HER2-enriched, basal-like, and normal breast-like [3]. The majority of basal-like breast cancers exhibit a triple-negative phenotype (ERα^−^, progesterone receptor-negative (PR^−^), Her2-neu-negative) and high frequency of p53 mutations [4]. Triple negative breast cancer is an aggressive histological subtype with poor prognosis and high rates of relapse following chemotherapy as compared to other subtypes [4, 5]. Nevertheless, studies of neoadjuvent chemotherapy suggest that women with TNBC who have a pathological complete response to treatment achieve excellent outcome (Carey LA 2007, Liedtke C 2008). Unfortunately, disease recurrence is very frequent, and conventional treatments for relapsed patients are limited. Therefore, there is an urgent unmet need for the development of novel generation of drugs with high efficiency and specificity against this particular group of patients.

ERα is a ligand-activated transcription factor, which plays major roles in breast carcinogenesis. Indeed, ERα signaling pathway is one of the most important pathways in hormone-dependent breast cancer. The amplification of the ERα coding gene *ESR1* is frequent in various breast tumors as well as in benign and precancerous breast diseases, suggesting that *ESR1* amplification may be a common mechanism in proliferative breast disease and a very early genetic alteration in a large subset of breast cancers [6]. Thereby, it’s reasonable to consider ERα inhibitors of significant clinical interest.

Several dietary phytochemicals have shown promising anti-cancer properties, and have been used as therapeutic agents against various illnesses for centuries [7]. Curcumin (diferuloylmethane), the major active component of the spice turmeric, has been widely used in traditional medicines for thousands of years [8]. Several in vitro and in vivo studies as well as clinical trials have shown that curcumin has potent anti-cancer effects, and safe even at high doses. However, curcumin exhibits poor aqueous solubility and low absorption in the gastrointestinal tract, which limits its clinical use [9]. To bypass this limitation, several curcumin analogues were synthesized, with the hope to increase the efficacy while preserving the same safety profile. PAC (4-hydroxy-3-methoxybenzylidene)-N-methyl-4-piperidone) is a promising anti-cancer curcumin analogue. Indeed, PAC is 5 times more efficient than curcumin in inducing apoptosis in breast cancer cells [10]. In the present study we have shown that PAC is more stable than curcumin in PBS and in circulating blood. Furthermore, PAC-dependent cytotoxicity is more potent on ERα^−^ cells than ERα^+^ cells through down-regulation of ERα. Moreover, PAC inhibits the pro-metastatic epithelial-to-mesenchymal transition (EMT) process in breast cancer cells, with higher efficiency on the TNBC subtype. Additionally, PAC suppressed the paracrine procarcinogenic effects of breast cancer cells on breast stromal fibroblasts via suppressing the secretion of two important cytokines IL-6 and MCP-1.

## Methods

### Ethics statement

Animal experiments were approved by the King Faisal Specialist Hospital and Research Center institutional Animal Care and Use Committee (ACUC) under the RAC proposal # 2080009, and were conducted according to relevant national and international guidelines. Animals suffered only needle injection pain and also certain degree of pain/distress related to the growth/ burden of the tumor. The euthanasia was performed using cervical dislocation.

### Cells and cell culture

The human breast cancer cell lines were all obtained from the American Type Culture Collection (ATCC). Cells were cultured following the instructions of the company. NBF-1 are primary normal breast fibroblasts developed from tissues obtained from plastic surgery, and cultured as previously described [11]. All supplements were obtained from Sigma (Saint Louis, MO, USA) except for antibiotic and antimycotic solutions, which were obtained from Gibco (Grand Island, NY, USA).

### Cellular lysate preparation and immunoblotting

This has been performed as previously described [12]. Antibodies directed against Vimentin (RV202), Twist1, N-cadherin and interleukin-6 (IL-6) were purchased from Abcam (Cambridge, MA); Akt, phospho-Akt (193H12), Erk1/2, phospho-Erk1/2 (THR202/TYR204), E-cadherin (24E10) and MCP-1 from Cell Signaling (Danvers, MA); c-Myc from BD Biosciences (San Jose, CA); Cyclin D1 (HD11), ERα (F-10) and glyceraldehydes-3-phosphate dehydrogenase (GAPDH, FL-335), were purchased from Santa Cruz (Santa Cruz, CA).

### RNA purification and quantitative RT-PCR

Total RNA was purified using the TRI reagent (Sigma) according to the manufacturer’s instructions, and was treated with RNase-free DNase before cDNA synthesis using the Advantage RT Kit (Clontech). For quantitative RT-PCR, the RT^2^ Real-Time™ SYBR Green qPCR mastermix (Qiagen, UK) was used and the amplifications were performed utilizing the Bio-Rad iQ5 multicolor Real time PCR detection system. The melting-curve data were collected to check PCR specificity, and the amount of PCR products was measured by threshold cycle (Ct) values and the relative ratio of specific genes to *GAPDH* for each sample was then calculated. The respective primers are:

- ***GAPDH***: 5’-GAGTCCACTGGCGTCTTC-3’ and 5’-GGGGTGCTAAGCAGTTGGT-3’;
- ***CCND1***: 5’ -TGTTCGTGGCCTCTAAGATGAAG-3’ and 5’- AGGTTCCACTTGAGCTTGTTCAC-3’
- ***c-MYC***: 5’- CTTCTCTCCGTCCTCGGATTCT-3’ and 5’-GAAGGTGATCCAGACTCTGACCTT-3’

### Transfection

The pGFP-C-shLenti plasmid bearing *ESR1*-shRNA or scrambled shRNA (Origene), were used at 1 μg/ml each for transfection of 293FT cells. Lentiviral supernatants were collected 48 h post-transfection. Culture media were removed from the target cells and replaced with the lentiviral supernatant and incubated for 24 h in the presence of 1 μg/ml polybrene (Sigma-Aldrich). Transduced cells were selected after 48 h with puromycin (Invitrogen).

### Bioavailability of PAC and curcumin

For the bioavailability experiments, normal Balb/c mice (*n* = 3×5, 25 g, 3 mice/time point) were intraperitoneally injected with PAC or curcumin (100 mg/Kg). 400 μL blood samples were withdrawn directly from the heart of each mouse into a heparin-rinsed vial at 15, 30, 45, 60 and 120 min post-injection. Each blood sample was centrifuged at 3000 × *g* for 5 min. The resulting plasma sample (100 μL) was acidified using hydrochloric acid (HCl 6 N, 10 μL) followed by three times extraction with a mixture of ethyl acetate:propanol (9:1, 1 mL). The extract was then completely dried and re-dissolved in methanol (100 μL) before direct injection onto HPLC for analysis. Using a standard curve of PAC and curcumin, the data obtained from analyzed samples were utilized to construct pharmacokinetic curve of PAC/curcumin concentrations in plasma using the Graph Pad Prism software. Separate experiments using the same extraction system were carried out to determine extraction efficiency of PAC and curcumin from plasma and showed a yield of 96 %.

### HPLC analyses

Reversed-phase HPLC were performed on Jasco, HPLC systems using C_18_ column (10 μm, 4.6 × 250 mm) (Econosil, Alltech, USA). HPLC was run using a gradient of 0.1 % TFA in water (solvent A) and 0.1 % TFA in CH_3_CN (solvent B) gradient, 0 to 50 % B, 15 min, 50 to 50 % B, 5 min, 50 to 0 % B, 5 min, and 100 to 100 % A, 5 min each at flow rate of 1 mL/min. The HPLC systems are equipped with a UV detector set at 420 nm, a Flow-count γ-radioactivity detection system (Bioscan, USA) and Lauralite analysis program (LabLogic, UK).

### Cell migration, invasion and proliferation

These assays were performed as described in detail previously [13]. Cell migration, invasion and proliferation were assessed in a real-time, non-invasive, and label-free manner using the xCELLigence RTCA technology (Roche). Migration and invasion were assessed as per the manufacturer’s instructions using the CIM-plate 16. Briefly, initially 160 μl of cell-free complete media, or SFM were added to the lower chamber wells and 30 μl of SFM to the upper chamber wells and plates were incubated for 1 h in the cell incubator to obtain equilibrium. Subsequently, the background signal was measured and exponentially growing cells resuspended in 100 μl of SFM were seeded in the upper chamber wells with a thin layer of matrigel basement membrane (invasion) or without (migration). Cells were seeded at 1–3 × 10^4^ cells/well. After cell addition CIM-plates 16 were incubated for 30 min at room temperature in the laminar flow. Subsequently, the plates were locked in the RTCA DP device in the incubator. Each condition was performed in triplicate.

For proliferation assays, exponentially growing cells in complete media (0.5–1 × 10^4^/well) were seeded in E-plates as per manufacturer’s instructions. The rest of the procedure was the same as for the invasion and migration assays. Relative cell migration, invasion and proliferation levels are shown in arbitrary units.

### Apoptosis analysis by annexin V/flow cytometry

Cells were either not treated or challenged with PAC or curcumin, and then harvested, centrifuged and stained with propidium iodide (PI) and Alexa Flour 488 annexin V (Molecular Probes, Eugene, OR, USA) as previously described [14].

### Protein arrays

SFCM were applied to RayBio the human cytokine array 5 (AAH-CYT-5, RayBiotech, Inc (Norcross, GA, USA) as per manufacturer’s instructions. Signal densities were assessed with the ImageJ software, and data analysis was carried out following the Array protocol’s instructions.

### Tumor xenografts

Breast cancer xenografts were created in nude mice by subcutaneous injection of MDA-MB-231 cells, and then the animals were treated with PAC (100 mg/Kg) or DMSO as previously described [10].

### Statistical analysis

Student’s *t*-test was used for statistical analysis and *p* values ≤ 0.05 were considered as statistically significant.

### Conditioned media

Cells were cultured in medium without serum for 24 h, and then media were collected and briefly centrifuged, and cells were counted. The resulting supernatants (SFCM) were used either immediately or were frozen at −80 °C until needed. SFCM were diluted, when necessary, based on the cell counts.

## Results

### PAC is more stable than curcumin in PBS and circulating blood

**Fig. 1:**
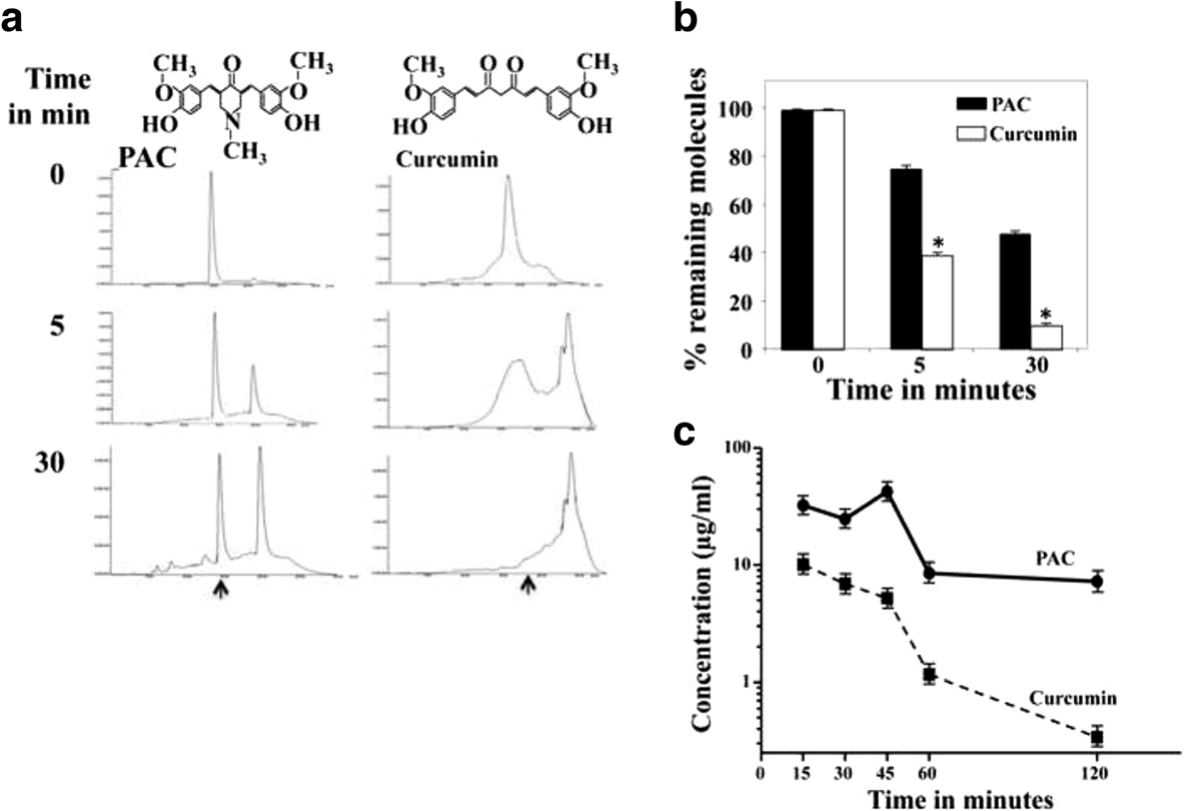
PAC is more stable than curcumin. **a** Curcumin and PAC structures. Both molecules were incubated in phosphate buffer (0.1 M, pH 7.4) for different periods of time, and then were analyzed by HPLC. The arrows indicate the peaks corresponding to the intact PAC and curcumin molecules. **b** Histogram showing the proportion of the remaining molecules. Error bars represent means ± SD from three different experiments, *, *P* ≤ 0.01. **c** Balb/c mice were intraperitonealy injected with PAC and curcumin (100 mg/Kg). Blood samples were withdrawn directly from the heart of each mouse at the indicated periods of time. The resulting plasma extracts were dried and re-dissolved in methanol before injection onto HPLC for analysis. The error bars represent means +/- SD

To compare the stability of curcumin with that of PAC, we have first studied the stability of both molecules in phosphate buffer (0.1 M, pH 7.4). PAC and curcumin were incubated in PBS for different periods of time (0, 5, 30 min), and then were analyzed by HPLC. Both PAC and curcumin were instable in PBS at 37 °C, but PAC showed higher stability (Fig. 1a). Indeed, after 30 min of incubation more than 90 % of curcumin was decomposed, while only 60 % of PAC was decomposed (Fig. 1b). This indicates that after 30 min of incubation PAC was 4 times more stable than curcumin in PBS.

In order to test the bioavailability of curcumin and PAC in animals, normal Balb/c mice (*n* = 30) were intraperitonealy injected with PAC or curcumin (100 mg/Kg). 400 μL blood samples were withdrawn directly from the heart of each mouse at 15, 30, 45, 60 and 120 min post-injection. The resulting plasma extracts were completely dried and re-dissolved in methanol (100 μL) before injection onto HPLC for analysis. Figure 1c shows that the amount of PAC presents in plasma at 15 min post-injection reached 35 μg/mL, while the level of curcumin was only 10.3 μg/mL. The level of PAC further increased in blood reaching its maximum level (40 μg/mL) 45 min post-injection, and dropped to 10 μg/mL at 60 min post-injection, then remained relatively constant during the following 60 min (Fig. 1c). On the other hand, the level of curcumin decreased in a time-dependent manner reaching a level as low as 0.3 μg/mL 120 min post-injection. This shows that 25 % of the injected PAC was still in blood 2 h post-injection, and that PAC is 30 times more stable than curcumin in circulating blood.

### PAC induces apoptosis in breast cancer cells with higher efficiency on basal-like cells

**Fig. 2:**
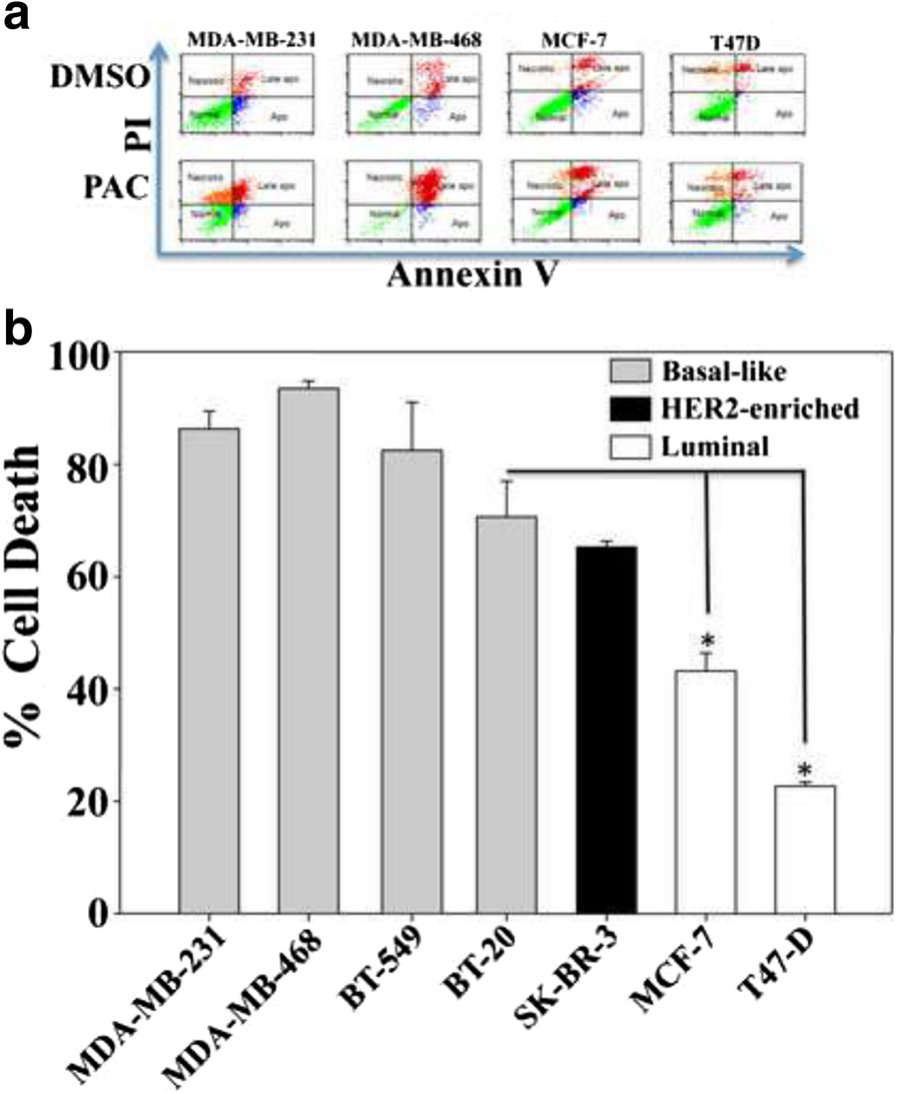
PAC triggers apoptosis more efficiently in ERα-negative than in ERα-positive breast cancer cells. Cells were either sham-treated or challenged with PAC (10 μM) for 72 h, and then cell death was assessed by annexin V/PI in association with flow cytometry. **a** Charts. **b** Histogram showing the proportion of cell death (apoptosis + necrosis) in each cell line. Error bars represent means ± SD from three different experiments, *, *P* ≤ 0.05

To measure the extent and the nature of cell death induced by PAC on various breast cancer cell lines, the fluorochrome-conjugated annexin V/PI stain test was used and cells were analyzed by a flow cytometer. Different cell lines were used including basal-like, ERα^−^ (MDA-MB-231, MDA-MB-468, BT-20, and BT-549), luminal, ERα^+^ (MCF-7, T-47D and BT-474), and HER2-enriched, ERα^−^ (SK-BR-3). Sub-confluent cells were treated either with DMSO (used as control) or with PAC (10 μM) for 72 h. Figure 2a shows the presence of four different cell populations after the double staining annexin V/PI and sorting by flow cytometry: live cells (normal) (annexin V-/PI-), early apoptotic cells (Apo) (annexin V+/PI-), late apoptotic cells (Late apo) (annexin V+/PI+) and necrotic cells (Necrotic) (annexin V-/PI+). Importantly, PAC induced cell death mainly by apoptosis in all breast cancer cells (Fig. 2a).

Figure 2b presents cell death of different cell lines after treatment with PAC as percentages relative to DMSO-treated cells. The real effect of PAC was obtained by discarding the proportion of dead cells obtained in the control samples from the treated ones for each cell line. In addition, the proportion of total cell death was considered as the sum of necrosis and both early and late apoptosis. 80–90 % of basal-like cells (MDA-MB-231, MDA-MB-468, BT-20, and BT-549) and about 65 % of HER2-enriched cells (SK-BR-3) underwent cell death in response to PAC (Fig. 2b). While in luminal cells, only 45 % of MCF-7 cells and 25 % of T-47D cells died upon PAC treatment (Fig. 2b). This indicates that PAC is cytotoxic against various types of breast cancer cells, and its effect is more potent on the ERα^−^ subtype.

### Specific ERα down-regulation sensitizes breast cancer cells to PAC

**Fig. 3:**
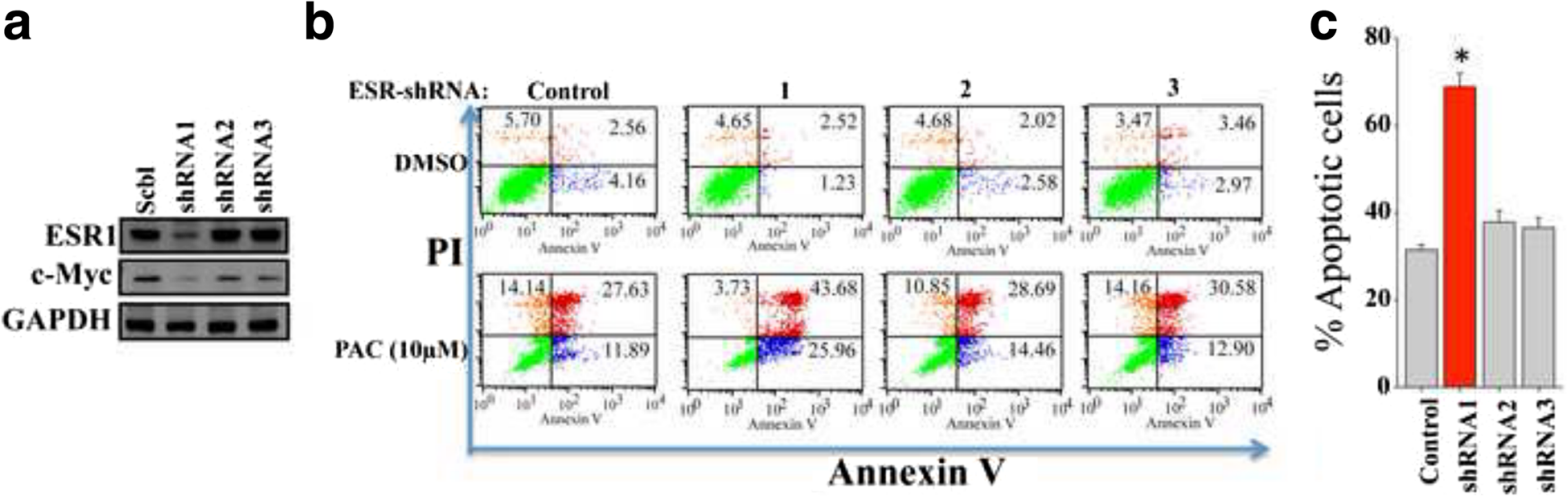
ERα down-regulation potentiates the pro-apoptotic effect of PAC. MCF-7 cells were transfected with vectors containing 3 different *ESR1*-shRNA sequences (1, 2, 3) or a scrambled sequence (Scbl). **a** Cell lysates were prepared and used for immunoblotting analysis using antibodies against the indicated proteins. **b** Cells were treated with PAC (10 μM) and the proportion of apoptotic cells was determined using annexinV/PI-Flow cytometry. **c** Histogram, Error bars represent means ± S.D from three different experiments, *, *P* ≤ 0.005

To explore the role of ERα in PAC-dependent induction of apoptosis, we knocked-down the *ESR1* gene in MCF-7 cells using specific *ESR1*-shRNA, and a scrambled sequence was used as control. Figure 3a shows strong ERα down-regulation by the *ESR1*-shRNA1, while *ESR1*-shRNA2 and *ESR1*-shRNA3 had only marginal effects. Similar effect was observed on the ERα target c-Myc (Fig. 3a). Subsequently, these cells were either sham-treated (DMSO) or challenged with PAC (10 μM) for 3 days, and then cell death was assessed by AnnexinV/PI as described above. Figure 3b and c shows that while PAC triggered apoptosis in only about 35 % of cells bearing control-shRNA, *ESR1*-shRNA2 or *ESR1*-shRNA3, the proportion of apoptotic cells reached 70 % in MCF-7 cells expressing *ESR1*-shRNA1. This shows that ERα down-regulation plays a major role in PAC-induced apoptosis in breast cancer cells.

### PAC down-regulates ERα, c-Myc and cyclin D1

**Fig. 4:**
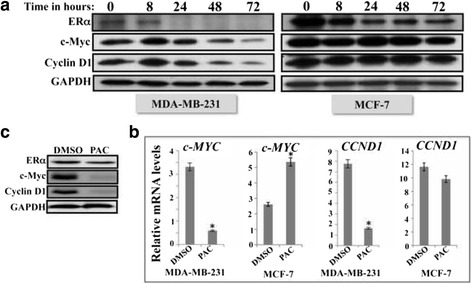
PAC down-regulates ERα and its targets C-Myc and cyclin D1. **a** Cells were challenged with PAC (10 μM) for the indicated periods of time, and then cell lysates were prepared and used for immunoblotting analysis utilizing specific antibodies against the indicated proteins. **b** Cells were either sham-treated (DMSO) or challenged with PAC (10 μM) for 24 h, and then total RNA was extracted and used for qRT-PCR using specific primers for the indicated genes. Error bars represent means ± S.D from three different experiments, *, *P* ≤ 0.05. **c** Nude mice bearing sub-cutaneous humanized tumor xenografts were treated with DMSO or PAC, and then cell lysates were prepared from excised tumors and used form immunoblotting utilizing specific antibodies against the indicated proteins

We then set out to test the effect of PAC on the expression of ERα in both types of cells (ERα -positive and –negative). To this end, MDA-MB-231 and MCF-7 cells were exposed to PAC (10 μM) and were harvested after different periods of time (0, 8, 24, 48, 72 h). Cell lysates were prepared and 100 μg/ml of proteins were used for immunoblotting analysis using specific anti- ERα antibody, and GAPDH was utilized as internal control. The level of ERα decreased in both cell lines, but became undetectable after 24 h of treatment in MDA-MB-231 cells, while a significant amount of the protein was still present in MCF-7 cells even after 72 h of treatment (Fig. 4a). The ERα protein is a well-known positive regulator of cyclin D1 and c-Myc, two important apoptosis modulators in breast cancer cells [15]. Therefore, we investigated the effect of PAC on the expression of these two proteins. Figure 4a shows that the levels of both proteins started to decrease after 24 h of treatment and continue to decline in a time-dependent manner in MDA-MB-231. On the other hand, the effect of PAC on cyclin D1 and c-Myc was only marginal in MCF-7 cells (Fig. 4a). To confirm this finding, we tested the effect of PAC on *CCND1* and *c-MYC* mRNAs. MDA-MB-231 and MCF-7 cells were either sham-treated (DMSO) or challenged with PAC (10 μM) for 24 h and total RNA was purified and used for amplification by quantitative RT-PCR using specific primers. Figure 4b shows that while PAC strongly down-regulated both genes in MDA-MB-231 cells, it increased the level of *c-MYC* and had only marginal effect on cyclin D1 in MCF-7 cells. This confirms the strong effect of PAC on ERα in ERα^−^ breast cancer cells and indicates that this gene as well as its targets *c-MYC* and *CCND1* might play important roles in the response of these cells to PAC. To further validate these findings we tested the effect of PAC on these three genes in tumor xenografts formed subcutaneously in nude mice upon injection of MDA-MB-231 [10]. The immunoblotting shows PAC-dependent down-regulation of the 3 genes in xenogratft tissues isolated from PAC-treated animals as compared to control animals treated with DMSO (Fig. 4c).

### PAC suppresses the epithelial-to-mesenchymal transition process both in vitro and in vivo

**Fig. 5:**
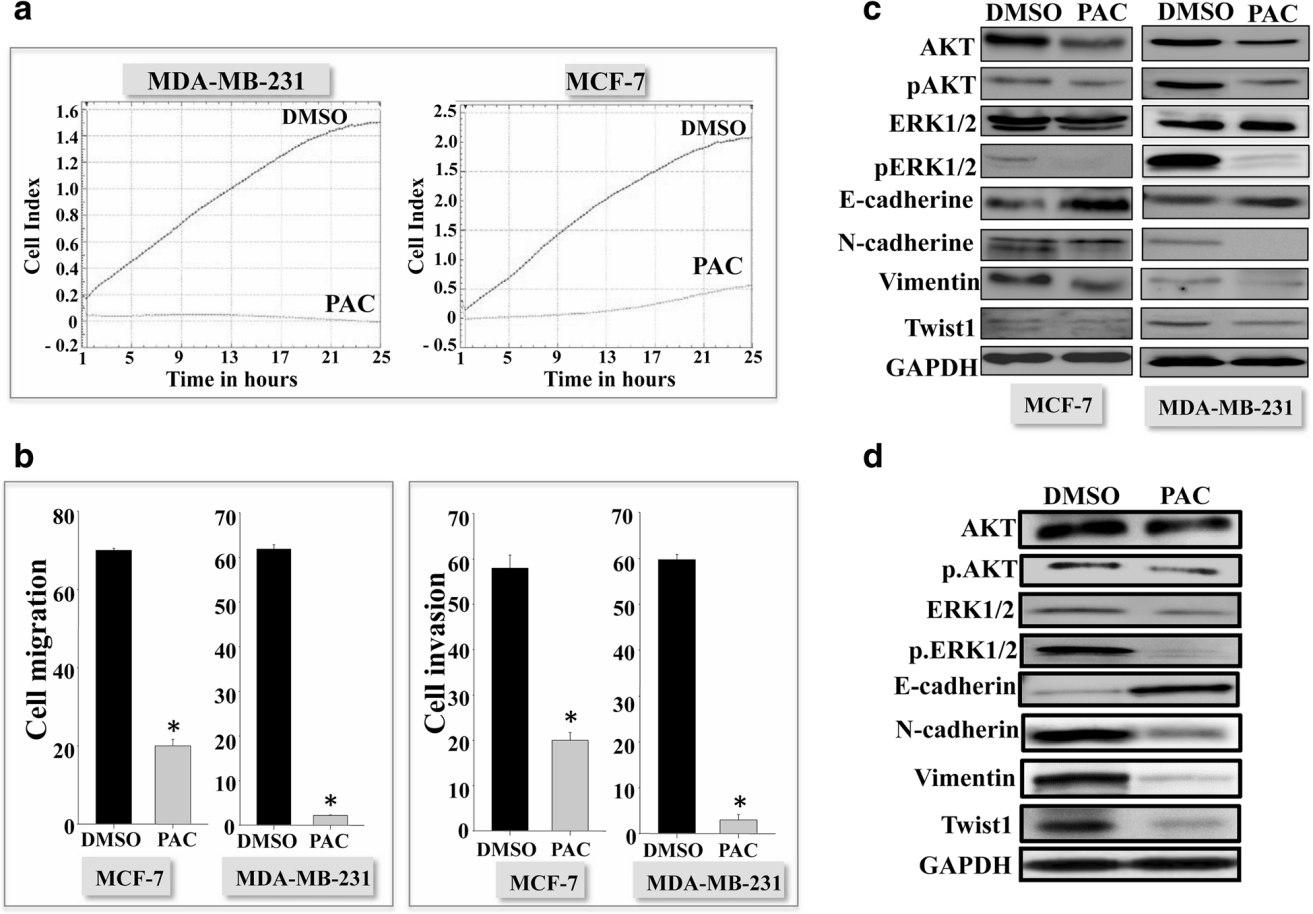
PAC suppresses EMT in breast cancer cells and tumor xenografts. **a** Cells were seeded in E-16 plates for 24 h, and then were either sham-treated (DMSO) or exposed to PAC (10 μM) for the indicated periods of time. Cell proliferation was monitored in real time using the RTCA-DP xCELLigence System. **b** Cells were exposed to DMSO or PAC (10 μM) for 24 h, and then were seeded in the CIM-plate with SFM in the upper wells separated by 8 micron pore size PET membrane with thin layer of matrigel basement membrane matrix (invasion) or without (migration). The lower chambers contained complete media as chemoattractant. Cells were incubated in normal culture conditions for 24 h and the migration/invasion were determined using the real time RTCA-DP xCELLigence System. Error bars represent means ± S.D from 3 different experiments; *, *P* ≤ 0.001. **c** Cells were either sham-treated (DMSO) or challenged with PAC (10 μM) for 24 h, and then cell lysates were prepared and used for Immunoblotting analysis using antibodies against the indicated proteins. **d** Figure legends as in Fig. 4c

Since estrogen receptor signaling plays a role in the pro-metastatic epithelial-to-mesenchymal transition (EMT) process, we sought to investigate the effect of PAC on EMT in both ERα^+^and ERα^−^ cells. To this end, MDA-MB231 and MCF-7 cells were either sham-treated or challenged with PAC (10 μM), and then cell proliferation was assessed using the xCELLigence RTCA technology utilizing the E-plates. Figure 5a shows strong PAC-dependent inhibition of cell proliferation of both MDA-MB-231 and MCF-7 cells, with a more pronounced effect on the ERα^−^ cells.

Next, we investigated the effect of PAC on the migration/invasion abilities using the xCELLigence RTCA technology utilizing CIM-plate 16. Figure 5b shows that PAC inhibits the migration and the invasion capabilities of both cell lines, with an effect more pronounced on the ERα^−^ MDA-MB-231 cells as compared to the ERα^+^ MCF-7 cells.

To elucidate the molecular basis of this decrease in the migration/invasion abilities, we tested the effect of PAC on the pro-invasive/migratory protein kinases ERK1/2 and AKT. Therefore, MDA-MB-231 and MCF-7 cells were either sham-treated or challenged with PAC (10 μM) for 24 h, and then cell lysates were prepared and total levels as well as the phosphorylated/active forms of these two proteins were assessed by immunoblotting. Figure 5c shows strong PAC-dependent inhibition of both protein kinases AKT and ERK1/2 in both cell lines, with a higher effect on MDA-MB-231 cells. Indeed, the inhibition of ERK1/2 was very strong in the TNBC cells (Fig. 5b). Since the activation of these two protein kinases is part of EMT, we investigated the possible PAC-dependent inhibition of this pro-metastatic process, through testing the effect on the expression of specific mesenchymal and epithelial markers by immunoblotting. Figure 5c shows clear PAC-dependent up-regulation of E-cadherin in MCF-7 cells, while the levels of all tested mesenchymal markers (N-cadherin, vimentin and Twist-1) were reduced in both cell lines, but the effect was more pronounced in MDA-MB-231 cells as compared to MCF-7 cells. Interestingly, similar results were obtained in vivo on tumor xenografts upon sub-cutaneous injection of MDA-MB231 cells in nude mice followed by intraperitoneal treatment with PAC (100 mg/Kg) (Fig. 5d). Indeed, PAC inhibited the two important protein kinases AKT and ERK1/2 and also up-regulated E-cadherin, while it down-regulated the mesenchymal markers N-cadherin, vimentin and twist1 (Fig. 5d). Together, these results indicate that PAC suppresses the EMT process in breast cancer cells both ERα -positive and -negative, with a more potent effect on ERα^-^ cells.

### PAC inhibits the expression/secretion of the pro-metastatic MCP-1 and IL-6 cytokines

**Fig. 6:**
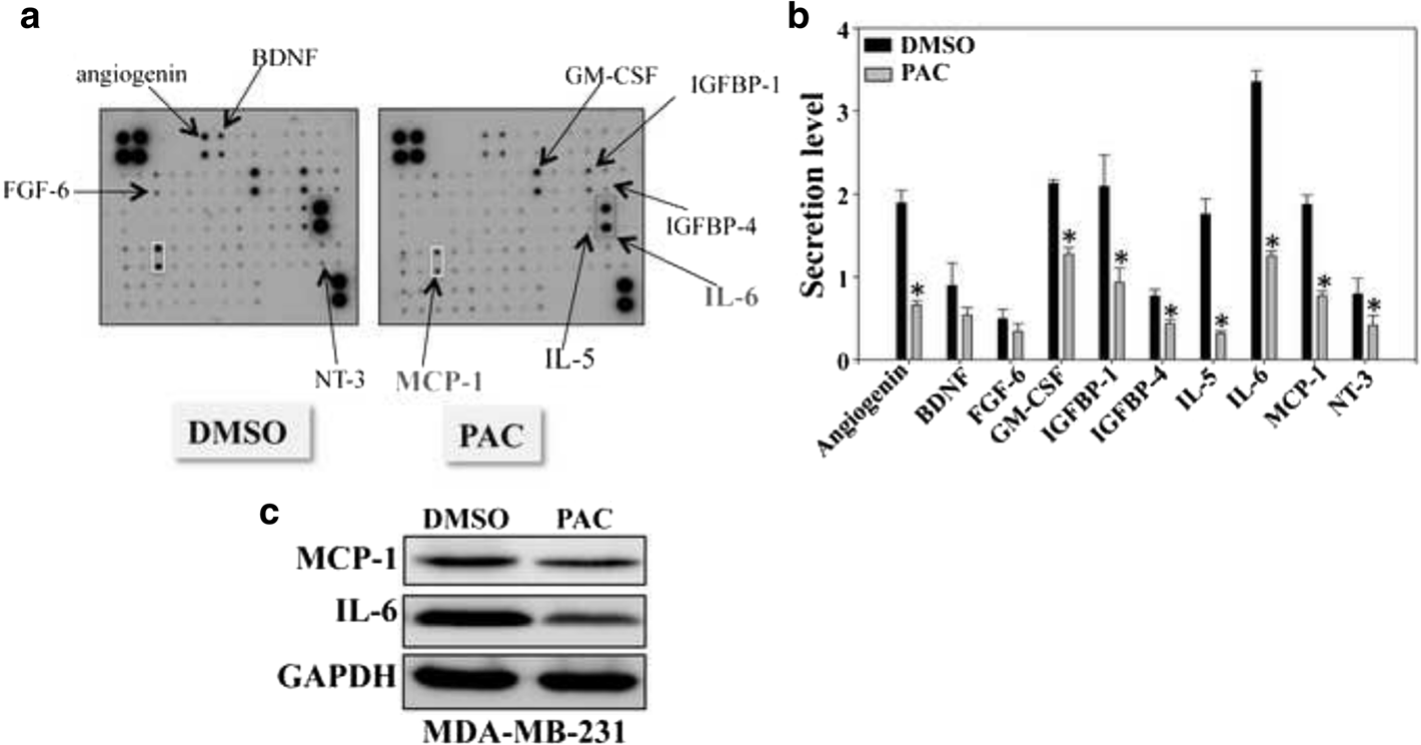
PAC inhibits the expression/secretion of IL-6 and MCP-1. SFCM from MDA-MB-231 cells either sham treated (DMSO) or challenged with PAC (10 μM) for 24 h were collected and applied onto Human Cytokine Antibody Array membrane. **a** Blots, **t**he rectangles indicate the spots corresponding to IL-6 and MCP-1, while the arrows indicate differentially expressed cytokines. **b** Histogram showing secretion levels of the indicated cytokines upon quantification. Error bars represent means ± S.D, *, *P* ≤ 0.05. **c** Cells were exposed to DMSO or PAC, and then cell lysates were prepared and used for immunoblotting analysis using specific antibodies against the indicated proteins

Human cytokine antibody arrays were used to detect the differentially expressed cytokines in the conditioned media obtained from MDA-MB-231 cells either sham-treated (DMSO) or challenged with PAC (10 μM). This experiment showed the differential expression of several cykokines, including IL-6, MCP-1, IL-5 and angiogenin (Fig. 6a and b). The effect on IL-6 and MCP-1 is of great importance since these two cytokines play important roles in the spread of various types of cancer, including breast carcinomas [16]. Therefore, we tested the effect of PAC on the expression of these two cytokines at the protein level. The immunoblotting analysis shows strong PAC-dependent down-regulation of both IL-6 and MCP-1 in MDA-MB-231 cells (Fig. 6c).

### PAC suppresses the paracrine effects of breast cancer cells on breast stromal fibroblasts

**Fig. 7:**
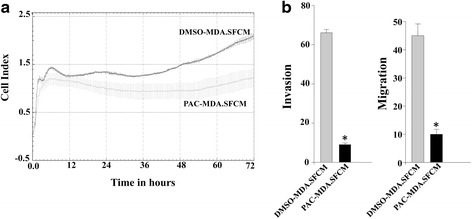
PAC suppresses the paracrine procarcinogenic effects of breast cancer cells. SFCM from MDA-MB-231 cells either exposed to DMSO (DMSO-MDA-SFCM) or challenged with PAC (10 μM) (PAC-MDA-SFCM) was used for indirect co-culturing of NBF-1 fibroblasts cells for 24 h, and then cell proliferation (**a**) as well as migration/invasion (**b**) were assessed using the real time RTCA-DP xCELLigence System as described in Fig. 4. Error bars represent means ± S.D from three different experiments; *, *P* ≤ 0.001

After showing PAC-dependent inhibition of IL-6/MCP-1 expression and secretion from breast cancer cells we sought to test the effect of PAC on the paracrine effects of these cells on breast stromal fibroblasts. To this end, MDA-MB-231 cells were first either sham-treated (DMSO) or challenged with PAC (10 μM) for 24 h. Subsequently, cells were cultured in serum-free medium (SFM) for 24 h, and then serum-free conditioned media (SFCM) were collected and utilized to challenge normal breast stromal fibroblast cells NBF-1 for 24 h. Proliferation, migration and invasion of stromal fibroblasts were assessed with the xCELLigence RTCA technology. Figure 7a shows PAC-dependent inhibition of the paracrine effects of MDA-MB-231 cells on the proliferation rate of fibroblast cells. Similarly, media conditioned with PAC-treated MDA-MB-231 cells strongly suppressed the migration/invasion abilities of breast stromal fibroblasts (Fig. 7b). Together, these results indicate that PAC inhibits the pro-metastatic effects of the highly invasive breast cancer cells MDA-MB-231.

## Discussion

Triple-negative breast cancers are poorly differentiated, highly malignant and have a poor outcome. Duration of response is usually short, with rapid relapse very common and median survival of advanced disease of just 13 months, which is much less than the median duration of survival observed in other advanced subtypes [17, 18]. This made TNBC one of the most attractive areas of research in oncology [19, 20].

We have shown here that the newly synthesized curcumin analogue PAC is 4 times more stable than curcumin in PBS and 30 times more stable than curcumin in circulating blood in mice (Fig. 1). This higher bioavailability of PAC is of great importance since the low bioavailability of curcumin limits its potential use in the clinic. Additionally, we present strong evidence that PAC is a potent anti-breast cancer agent with the strongest effects on the TNBC subtype cells. Indeed, PAC exhibited higher cytotoxicity against different TNBC cells (MDA-MB-231, MDA-MB-468, BT-20 and BT-549) than luminal cells (MCF-7 and T-47D). Likewise, PAC inhibited the proliferation of breast cancer cells with higher effect on ERα^−^cells than on ERα^+^ cells. Interestingly, when ERα was down-regulated in MCF-7 cells with specific shRNA, the pro-apoptotic effect of PAC was doubled. This clearly showed that high expression of ERα limited PAC cytotoxic effects on breast cancer cells, and we hypothesized that PAC-dependent apoptosis could be mediated through ERα down-regulation. Therefore, we tested the effect of PAC on the expression of ERα in cells that express high and low level of this receptor, and have shown that PAC reduced ERα level in both types of cells, but in the ERα^−^ cells ERα became undetectable, while its level was still high in ERα^+^ cells despite its diminution. This provided a meaningful explanation to the higher effect of PAC on ERα^−^cells, and corroborated the result obtained by specific down-regulation of ERα in cells that express this receptor. This also confirmed our previous results showing that expression of the receptor coding gene *ESR1* in ERα^−^ breast cancer cells increased their resistance to PAC [10]. Together, these results indicate that PAC-related induction of apoptosis in breast cancer cells depends on the cellular level of ERα. In fact, various laboratory studies demonstrated that reduced estrogen levels induced apoptosis [21]. A cyclopentenone derivative (CTC-35) was also shown to have potent proapoptotic activity in ERα^−^ breast cancer cells [22]. Furthermore, green tea EGCG decreased the proliferation rates of breast cancer cells through strong down-regulation of ERα [23].

PAC-dependent down-regulation of ERα in MDA-MB-231 cells was accompanied with a strong decrease in the level of ERα main targets c-Myc and cyclin D1, both in vitro and in vivo. However, c-Myc and cyclin D1 were not down-regulated in MCF-7 cells, and the c-MYC mRNA was rather up-regulated after 24 h of PAC treatment (Fig. 4b). This difference in the c-MYC mRNA and protein levels upon PAC treatment could result from translational of post-translational regulatory process in MCF-7 cells. c-MYC and cyclin D1 are two major oncogenes, which confer proliferative and anti-apoptosis capacities to breast cancer cells [15], and are associated with altered sensitivity to endocrine therapy [24]. The c-Myc protein plays a major role in the apoptotic response of breast cancer cells [25]. This protein has been found overexpressed in 45 % breast tumors [26]. Cyclin D1 is an oncogene that is overexpressed in about 50 % of all breast cancer cases [27], and its down-regulation is an important target in breast cancer therapy [28]. Therefore, PAC-related targeting of these two oncogenes could be of great therapeutic value.

In addition, we have shown that PAC suppresses the EMT process in both ERα^+^ and ERα^−^ cell lines, with a higher effect on ERα-negative cells (Fig. 5). EMT is currently considered as pivotal event in the initial step of the metastatic cascade that allows cells to acquire migratory, invasive and stem-like properties [29]. Evolving evidence indicates that ERα signaling can directly regulate EMT-related transcriptional factors, indicating that ERα might be a key regulator of the EMT program [30–33]. PAC inhibited the migration/invasion abilities of breast cancer cells through inhibiting the ERK1/2 and AKT protein kinases, and repressed the mesenchymal markers vimentin and N-cadherin. Similar effects were also observed in vivo on tumor xenografts, wherein PAC increased the expression of E-cadherin and repressed N-cadherin, vimentin, AKT and Twist1. Similarly, it has been previously shown that curcumin plays an important role in the inhibition of lipopolysaccharide-induced EMT in breast cancer cells through the down-regulation of NF-kB-Snail activity [34].

In addition to their high migratory and invasiveness capacities, ERα^−^ cells secrete high amounts of pro-metastatic cytokines such as IL-6 and MCP-1, which can activate stromal cells including fibroblasts [16, 35, 36]. Interestingly, PAC reduced the secreted levels of several cytokines including IL-6 and MCP-1 from MDA-MB-231 cells. This repressed the paracrine pro-replicative and -invasive/migratory effects of these highly invasive cells on breast stromal fibroblasts. This shows the ability of PAC in inhibiting the pro-metastatic capabilities of MDA-MB-231 cells. Indeed, there exists abundant evidence demonstrating that active stromal fibroblasts play major roles in breast cancer progression and spread [37]. These cells escort cancer cells through the whole carcinogenesis process. Therefore, targeting active stromal fibroblasts or blocking their cross-talk with cancer cells is a promising therapeutic approach [38, 39].

## Conclusions

We have shown here that PAC has better bioavailability than curcumin. Moreover, we present clear evidence that PAC down-regulates ERα and triggers apoptosis in breast cancer cells with higher efficiency on receptor negative cells. Furthermore, PAC suppressed the prometastatic features of the invasive breast cancer cells by suppressing EMT and the paracrine effect on breast stromal fibroblasts. This makes PAC as a valuable candidate for the future armada against the devastating TNBC type of tumors.

## Abbreviations

ATCC, American type culture collection; DMSO, dimethyl sulfoxide; EMT, epithelial-to-mesenchymal transition; ERα, estrogen receptor alfa; GAPDH, glyceraldehyde-3-phosphate dehydrogenase; PBS, phosphate buffered saline; RT-PCR, reverse transcriptase-polymerase chain reaction; shRNA, short hairpin RNA
